# The pathophysiology of pelvic ring injuries: a review

**DOI:** 10.1186/s13037-024-00396-x

**Published:** 2024-05-13

**Authors:** Philip F. Stahel, Navid Ziran

**Affiliations:** 1 Department of Surgery, East Carolina University, Brody School of Medicine, 27834 Greenville, NC USA; 2 Rocky Vista University, College of Osteopathic Medicine, 80134 Parker, CO USA; 3 Mission Health, HCA Healthcare, North Carolina Division, 28803 Asheville, NC USA; 4 St. Joseph’s Hospital and Medical Center, 85020 Phoenix, AZ USA; 5 North Bay Medical Center, 94534 Fairfield, CA USA; 6 Satori Orthopaedics, Inc, 85020 Phoenix, AZ USA

**Keywords:** Pelvic ring injury, Pathophysiology, Retroperitoneal hemorrhage, Lethal triad, Coagulopathy

## Abstract

Traumatic pelvic ring injuries continue to represent a major challenge due to the high rates of post-injury mortality of around 30-40% in the peer-reviewed literature. The main root cause of potentially preventable mortality relates to the delayed recognition of the extent of retroperitoneal hemorrhage and post-injury coagulopathy. The understanding of the underlying pathophysiology of pelvic trauma is predicated by classification systems for grading of injury mechanism and risk stratification for developing post-injury coagulopathy with subsequent uncontrolled exsanguinating hemorrhage. This review article elaborates on the current understanding of the pathophysiology of severe pelvic trauma with a focus on the underlying mechanisms of retroperitoneal bleeding and associated adverse outcomes.

## Introduction

Traumatic disruptions of the pelvic ring result from high-energy trauma mechanisms and represent a major source of life-threatening hemorrhage and potentially preventable mortality in young trauma patients [1–3]. Physiologic instability can also result from low-energy pelvic ring injuries in elderly patients with poor quality bone stock [4]. The main root cause of the high mortality around 30-40% in the modern literature is due to the underrecognized presence of exsanguinating hemorrhage and “hidden shock” from occult bleeding sources in the retroperitoneal space [5–9]. The pelvic bowl contains about 1,500 cc in volume, which increases exponentially in in patients with mechanically unstable pelvic ring injuries [10]. Experimental cadaveric studies revealed that the pelvic volume increases around 20% in presence of a pubic symphysis disruption of 5 cm, and up to 40% with a pubic diastasis of 10 cm [10, 11]. Patients who survive acute pelvic ring injuries are often confronted with long-term rehabilitation and residual functional impairment related to gait and mobility, associated urogenital and neurological injuries, sexual impairment, and chronic pain [12–14]. The traumatic hemorrhage in high-energy pelvic ring disruptions relates in large part to venous bleeding sources in the retroperitoneal space (> 90%) and rarely to arterial bleeding sources (< 10%) [15–18]. The main pelvic bleeding sources originate from extensive retroperitoneal plexuses and cancellous bone bleeding from the posterior pelvic elements, including sacral fractures and iliosacral joint disruptions [19, 20]. In addition, about one third of all patients with traumatic pelvic ring disruptions are coagulopathic on admission which exacerbates the extent of traumatic pelvic hemorrhage [21, 22]. This review article was designed to elaborate on the pathophysiology of severe pelvic trauma with a focus on the underlying mechanisms of retroperitoneal bleeding and associated adverse outcomes.

### Injury mechanism

The mechanism of injury represents a crucial early screening tool to identify patients “at risk” for pelvic ring disruptions and associated traumatic hemorrhage [23]. Most pelvic ring injuries are caused by blunt trauma forces related to deceleration mechanisms from motor vehicle or motorcycle accidents and falls from heights [24]. The American College of Surgeons Committee of Trauma (ACS-COT) defines a threshold of 6 m (20 ft) as a critical falling height predictive of the potential for sustaining major injuries [25]. While falls from higher than 100 ft are considered “non-survivable” occasional case reports have described survival after a free fall from 300 ft height [26]. Of academic interest only, the highest recorded falling height survival is attributed to the Serbian flight attendant Vesna Vulović who survived a plane crash at 33,330 feet or 10.16 km (6.31 miles) after a bomb exploded on JAT Airways 367 on January 26, 1972. While all other crew members and passengers fell to their deaths after being blown out of the exploding aircraft once the cabin depressurized, Vesna Vulović’s survival is attributed to her being trapped by a food trolley and surviving the fall in the airplane’s broken fuselage.

The exact injury mechanism in conjunction with the overall injury severity, as defined by trauma scoring systems, and the patient’s physiological response to resuscitation represent decisive variables predictive of survival [27]. The Advanced Trauma Life Support (ATLS) protocol provides a pragmatic stratification of the extent of traumatic hemorrhage and the associated changes in clinical presentation (Table 1) [25].

**Fig. 1:**
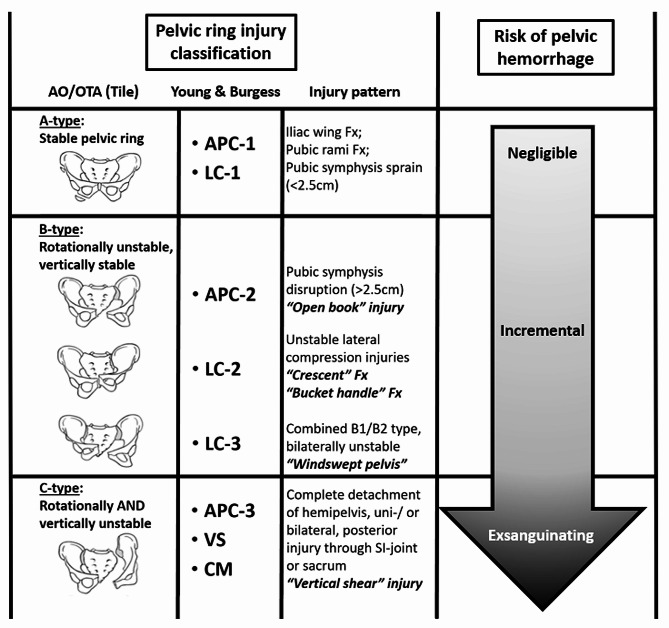
Pelvic ring injury classification, severity grading, and risk stratification for associated traumatic hemorrhage. Abbreviations: APC, antero-posterior compression; AO, Arbeitsgemeinschaft für Osteosynthesefragen; CM, combined mechanism, LC, lateral compression; OTA, Orthopaedic Trauma Association; VS, vertical shear

**Fig. 2:**
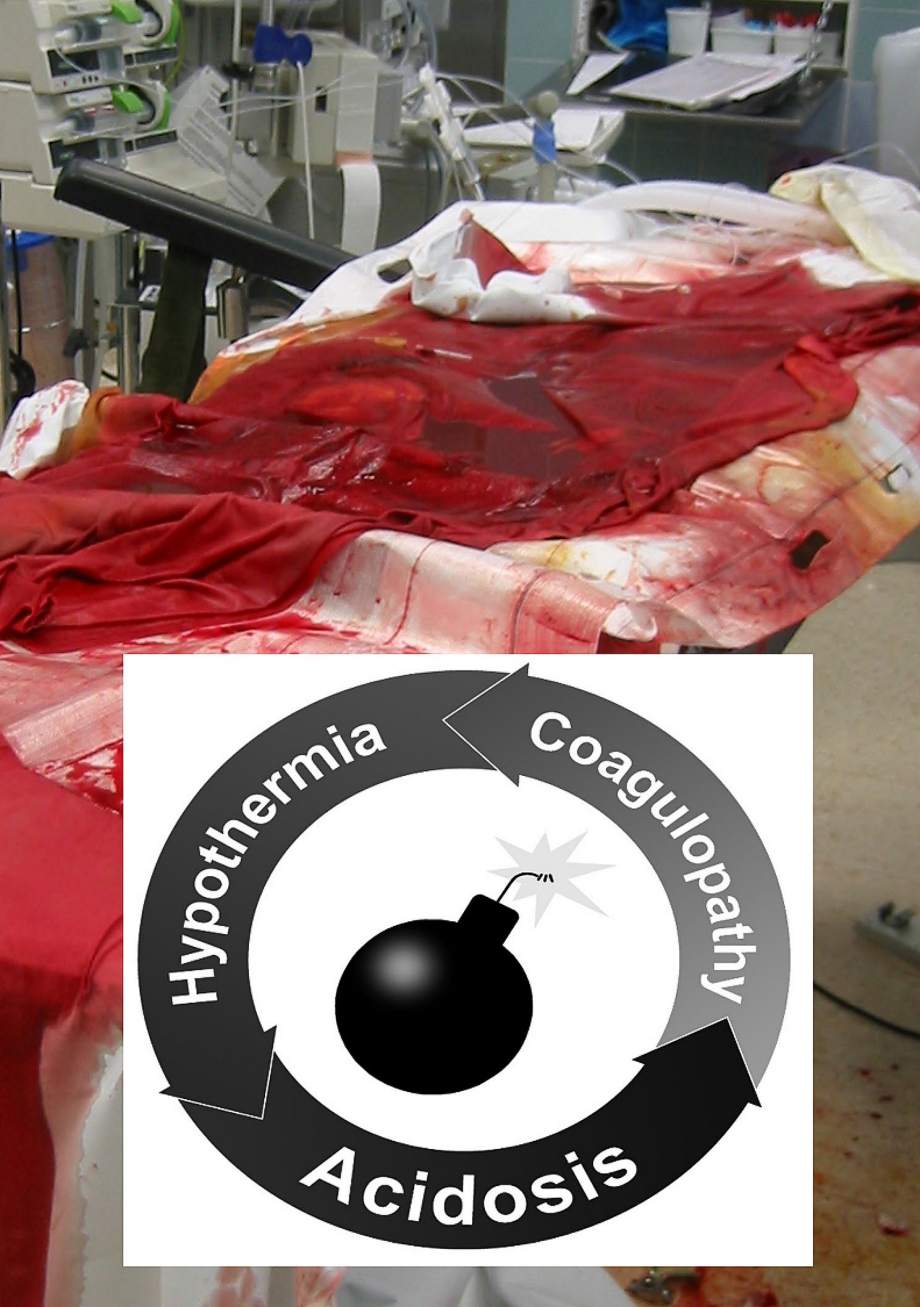
The lethal triad of traumatic hemorrhage

The biomechanical stability of the pelvic ring relies on the integrity of the pubic symphysis and the posterior ligamentous complex [23]. With increasing impacting force, a partial or complete disruption of the iliosacral ligaments leads to a critical amount of retroperitoneal bleeding and potentially life-threatening hemorrhagic shock [28]. The vector of the impacting force has been shown to drive specific patterns of pelvic ring disruptions and determine their underlying extent of biomechanical instability and risk of associated bleeding. Prevalent classification systems, including the alpha-numeric AO/OTA (Tile) classification and the mechanistic classification by Young & Burgess, are essentially based on the direction and extent of the impacting force onto the pelvic ring (Fig. 1) [29, 30]. As such, antero-posterior compression (APC) mechanisms induce an incremental disruption of the pubic symphysis with an external rotation deformity of the injured hemipelvis (“open book”) and consecutive hinging/tensile forces on the iliosacral ligaments [31]. In contrast, lateral compression (LC) injuries lead to an internal rotation deformity of the injured hemipelvis with incremental disruption of the iliosacral ligament complex by compressing forces [31]. Finally, the “vertical shear” (VS) and “combined mechanism” (CM) injury patterns are sustained by massive axial loading forces, including high-speed acceleration/deceleration collisions and falls from significant heights, leading to a complete disruption of the pelvic ring integrity, with external rotation and vertical translation of the injured hemipelvis [31]. The VS and CM type injuries are invariably associated with acute life-threatening exsanguinating hemorrhage [20]. Therefore, in patients with hemodynamically unstable pelvic ring injuries, the early recognition and mitigation of the “lethal triad” of metabolic acidosis, hypothermia, and coagulopathy represents the key determinant for patient survival (Fig. 2) [27].

**Fig. 3:**
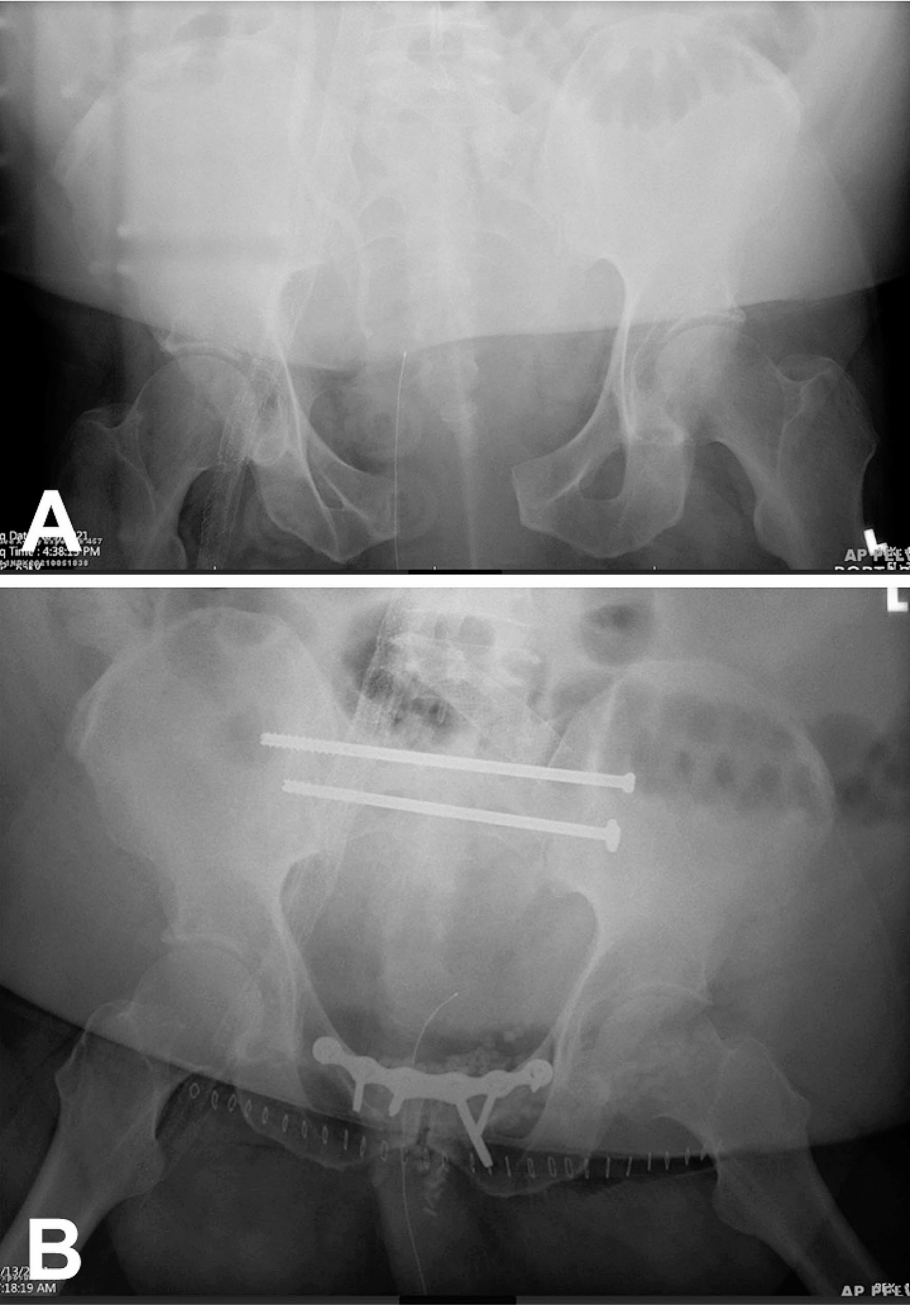
Clinical case scenario of a patient with life-threatening exsanguinating hemorrhage associated with a high-energy pelvic ring disruption

### “Hidden shock”

Circulatory compromise in patients with pelvic ring injuries can be challenging to recognize during the early stages of pelvic hemorrhage in young patients who can remain clinically compensated in spite of significant pelvic hemorrhage (Table 1) [32]. Therefore, normotensive patients with pelvic ring injuries are considered to be in a state of “hidden shock” from potential retroperitoneal blood loss until proven otherwise [16]. The question of whether a trauma patient is in “hidden shock” is addressed by clinical parameters, laboratory testing, and imaging studies, as outlined further below. The bleeding trauma patient’s oxygen requirement is illustrated by the historic Nunn & Freeman formula from 1964: *O2*_*av*_ *= CO × SaO*_*2*_*× Hb × 1.34* [33]. This equation clarifies that the available tissue oxygen (O2_av_) is equal to the product of cardiac output (CO in ml/min), arterial oxygen saturation (SaO_2_ in %) and hemoglobin concentration (Hb in g%), whereby the Nunn-Freeman constant of 1.34 represents the O2-binding capacity of hemoglobin (in ml/g) [33]. While the oxygen demand and supply is generally met under physiological conditions, the underlying variables of the Nunn-Freeman formula are dramatically compromised in multiply injured patients due to acute blood loss (Hb), pulmonary contusions (SaO_2_), and myocardial contusion or pericardial tamponade (CO), resulting in a limiting deficit of systemic oxygen supply [33].

### Clinical windows to the microcirculation

As part of the clinical exam, there are selected diagnostic “windows” into the microcirculation which allow estimating the trauma patient’s state of shock [25]. These include pulse examination for heart rate (tachycardia) and amplitude, skin perfusion for hypovolemia, level of consciousness as a surrogate of cerebral perfusion, and urinary output for assessment of renal organ perfusion [25].

1. Skin perfusion: Patients with pink skin in the face and extremities are likely not at risk of significant hypovolemia. In contrast, the presence of cold and clammy skin, with ashen-grey facial skin, pale extremities, and delayed capillary refill in conjunction with tachycardia are strong clinical indicators of traumatic-hemorrhagic shock.
2. Cerebral perfusion: When the circulating volume is critically reduced due to hypovolemia, patients may present with an altered level of consciousness due to cerebral hypoperfusion. However, this may represent a late sign of significant hemorrhage due to the physiological autoregulation which retains cerebral blood flow in presence of systemic hypotension. Agitation, confusion, somnolence or lethargy may represent indirect signs of critical cerebral hypoperfusion in bleeding trauma patients.
3. Renal perfusion: The placement of a Foley catheter allows to monitor the extent of urine production as a surrogate marker of renal perfusion. Patients with severe hypovolemia will present with oliguria (defined as < 0.5 ml/Kg BW/hr) or anuria. The Foley catheter furthermore allows to detect macrohematuria secondary to renal trauma or urogenital injuries.

In order to estimate the approximate extent of traumatic hemorrhage, the compensatory mechanisms to hypovolemia and response to resuscitative measures have to be taken into consideration [16]. For example, the acute blood loss of up to 30% of the circulating volume (equivalent to about 1,500 cc in an average patient of 70 kg body weight) does not lead to hypotension due to the increase in peripheral resistance, in spite of a significant reduction in cardiac output (Table 1) [25]. The clinical “windows” and response to resuscitation provide a rough estimate to determine if a trauma patient with pelvic ring injury is “hemodynamically normal” or just apparently and transiently “hemodynamically stable” [25]. Based on the response to resuscitative measures, patients are stratified into “responders”, “non-responders”, and “transient responders” [25]. The latter cohort of patients are frequently under-triaged due to occult hemorrhagic shock, with a high risk of acute deterioration and preventable adverse outcomes [34].

### Laboratory testing

A complete blood count (CBC) represents a part of the baseline diagnostic work-up for trauma patients [34]. However, the diagnostic value of hemoglobin or hematocrit for occult hemorrhage in trauma patients remains a topic of debate [34]. One major drawback of isolated hemoglobin or hematocrit values is due to the confounding influence of dilution by administration of crystalloids [34]. Recent studies have unequivocally determined that neither isolated nor serial repeat assessment of hemoglobin or hematocrit represent sensitive tests to predict the necessity for emergent surgical intervention in blunt trauma patients with occult hemorrhage [35]. In contrast to the poor predictive value of the CBC, both base deficit and serum lactate have been shown to significantly predict the presence of “hidden shock” in trauma patients and to monitor the response to resuscitation [35]. The extent of shock by base deficit is stratified into 3 categories: mild (-3 to -5 mEq/l), moderate (-6 to -9 mEq/l) and severe (<-10 mEq/l) [35]. This stratification provides a significant correlation between the admission base deficit and transfusion requirements within the first 24 h and the risk of postinjury complications and death [35]. It is also important to note that the base deficit is a better prognostic marker of death than the pH, by arterial blood gas analysis [35]. The base deficit has been established as a highly sensitive marker for the extent of post-traumatic shock and mortality, both in adult and paediatric patients [35]. In essence, a base deficit below − 5 mEq/l by arterial blood gas analysis is associated with a significantly increased rate of postinjury complications and transfusion requirements, whereas a level less than − 10 mEq/l is associated with a very high predicted mortality [35]. In contrast, a normal base deficit (or base excess) with values around + 2 to − 2 mEq/l is associated with a low postinjury mortality of < 10% [35].

Historic landmark studies have shown that the serum lactate level on admission represents a “key” predictor for the presence of traumatic-hemorrhagic shock on admission [36, 37]. Abramson and colleagues performed a prospective observational study in patients with multiple trauma to evaluate the correlation between lactate clearance and survival [36]. All patients in whom lactate levels returned to the normal range (≤ 2 mmol/l) within 24 h survived [36]. Survival decreased to 77.8% if normalisation occurred within 48 h and to 13.6% in those patients in whom lactate levels were elevated above 2 mmol/l for more than 48 h [36]. These findings were confirmed in a study by Manikis and colleagues who showed that the initial lactate levels were higher in non-survivors after major trauma, and that the prolonged time for normalisation of lactate levels of more than 24 h was associated with the development of post-traumatic organ failure [37].

Although both the base deficit and serum lactate levels are well correlated with the extent of traumatic-hemorrhagic shock and response to resuscitation, these two parameters do not strictly correlate [38]. Therefore, the independent assessment of both parameters is recommended for the initial evaluation of the bleeding trauma patient [35].

### Postinjury coagulopathy

Uncontrolled hemorrhage accounts for the high mortality in patients with pelvic ring disruptions of which around one third present with coagulopathy on admission [39]. This subset of trauma patients has a significantly increased risk of adverse outcomes and death compared to non-coagulopathic patients with similar injury severity [40]. The diagnostic workup for postinjury coagulopathy includes conventional laboratory tests, such as the international normalised ratio (INR), activated partial thromboplastin time (aPTT), fibrinogen levels and platelet count [34]. In general, the diagnosis of coagulopathy using conventional assays is determined by the following thresholds [34]:

- Prothrombin time (PT) > 18 s.
- Activated partial thromboplastin time (aPTT) > 60 s.
- PT/aPTT > 1.5x control values.
- INR > 1.5 (PT).
- Quick value < 70% (PT).
- Platelet count < 100 × 10^9^/L.

However, most of the conventional coagulation tests were developed to monitor anticoagulant therapy, and therefore reflect a crude and artificial in vitro assessment of coagulation [21, 41, 42]. The pure reliance on in vitro coagulation tests (which are performed at a normal pH and a temperature of 37 °C) does not reflect the “true” in vivo coagulopathy in hypothermic and acidotic trauma patients [16]. In addition, the testing by conventional coagulation parameters is associated with a significant delay of around 20–30 min until results are available, and the patient’s state of coagulopathy will have changed by the time results are available, due to ongoing resuscitation efforts [27].

These significant limitations of conventional laboratory tests are mitigated by modern “point of care” coagulation assays, using thromboelastography (TEG) or rotational thromboelastometry (ROTEM) [39, 42–44]. These modalities are performed quickly at the bedside, and thus represent a “real-time” assessment of coagulation in the bleeding trauma patient.

### Risk stratification

The international consensus guidelines by the *World Society of Emergency Surgery* (WSES) furthermore provides a classification system for risk stratification of patients with pelvic ring injuries and associated hemorrhage [45]. The WSES system takes into account the mechanical stability of the pelvic ring in conjunction with hemodynamic stability based on the established ATLS® criteria [25, 46].

1. **Grade 1 (Minor)**.

Mechanically and hemodynamically stable pelvic ring injury patterns (APC-1, LC-1).

2. **Grade 2 (Moderate)**.

Rotationally unstable pelvic ring injuries (LC-2, APC-2) with hemodynamic stability and/or adequate response to resuscitation (“responders“).

3. **Grade 3 (Moderate)**.

Rotationally and vertically unstable pelvic ring injuries (APC-3, LC-3, VS, CM) with hemodynamic stability and/or adequate response to resuscitation (“responders“).

4. **Grade 4 (Severe)**.

*Any* mechanically unstable and hemodynamically unstable injury pelvic ring injury pattern at risk for fatal outcome from acute exsanguinating hemorrhage (“non-responders“).

Another pragmatic approach for timely decision-making regarding the optimal treatment modality of pelvic ring injuries and associated hemorrhage is represented by the simplistic risk stratification into the cohorts *stable/borderline/unstable/in extremis* based on their physiological status and response to resuscitation [20, 27, 47].

### Stable

Patients classified as stable typically respond to the initial treatment and remain hemodynamically stable without clinical or laboratory signs of occult hemorrhage and “hidden shock.”

### Borderline / “at risk”

A persistent base deficit, elevated lactate levels, and abnormal coagulation measures in patients with pelvic ring injuries are indicative of persistent “hidden shock” and ongoing resuscitation requirements. These trauma patients typically present with a combination of injury patterns that renders them at risk of adverse outcomes. The patients may be under-triaged due to initial response to resuscitation (“transient responders”) with rapid subsequent deterioration.

Criteria for identifying “at risk” borderline patients (with or without pelvic ring injuries) include [27, 48, 49]:

- Hypothermia (< 36ºC).
- Acidosis (lactate, BD).
- Coagulopathy (INR, aPTT, TEG/ROTEM).
- Severe traumatic brain injury (GCS ≤ 8).
- Bilateral femur shaft fractures.
- Radiographic evidence of pulmonary contusions.
- Multiple injuries in association with thoracic trauma or head injury.
- Multiple injuries in association with severe abdominal or pelvic trauma.

### Unstable

This subset of critically injured patients present with hypotension (systolic BP < 90mmHg) with signs of traumatic-hemorrhagic shock grade 3 or grade 4 (Table 1). “Non-responders” and “transient responders” typically require immediate life-saving surgery and timely transfer to ICU for restoration of the “endpoints of resuscitation” (see below).

### In extremis

These patients present in a state of uncontrollable exsanguinating hemorrhage and have a high predicted mortality. These patients are non-responders by definition, and require immediate activation of a mass transfusion protocol (MTP) in conjunction with “damage control” procedures at the bedside, including ED thoracotomy and “crash” laparotomy [50]. Once the life-saving procedures are carried out, patients are transferred directly to ICU for monitoring and ongoing resuscitation [27].

## Conclusion

The pathophysiology of pelvic ring injuries with associated retroperitoneal hemorrhage is predicated by the specific injury mechanism and classification-based injury severity. The “key” objective in the workup and management of these critical injuries relies on the appropriate risk stratification based on the underlying pathophysiology of associated pelvic hemorrhage and coagulopathy, with the goal of restoring the normal physiology based on the following end-points of resuscitation [27]:

- Stable hemodynamics, without the need for vasoactive or inotropic stimulation.
- Absence of hypoxemia or hypercapnia.
- Serum lactate < 2.5 mmol/L.
- Normal coagulation (INR, TEG/ROTEM).
- Normothermia (> 36 °C / 96.8 °F).
- Normal urinary output (> 1 ml/Kg BW/hr).

The specific management strategies of hemodynamically unstable pelvic ring injuries is beyond the scope of this review and described elsewhere in the published literature [51–55].

**Table 1:** Classification of traumatic-hemorrhagic shock*

	Class 1	Class 2	Class 3	Class 4
Blood loss	< 750 cc	750-1,500 cc	1,500-2,000 cc	> 2,000 cc
Blood loss (% volume)	< 15%	15-40%	30-50%	> 40%
Heart rate	< 100/min	> 100/min	> 130/min	> 140/min
Blood pressure	Normal	Normal	Decreased	Decreased
Pulse pressure	Normal	Decreased	Decreased	Decreased
Respiratory rate	14–20/min	20–30/min	30–40/min	> 35/min
Urine output	> 30mL/hr	20–30/mL/hr	5–15/ml/hr	Negligible
Mental status	Normal	Anxious	Confused	Lethargic

*per ATLS® criteria [25]

## Data Availability

No datasets were generated or analysed during the current study.
